# Therapeutic drug monitoring of amikacin in septic patients

**DOI:** 10.1186/cc12844

**Published:** 2013-07-25

**Authors:** Wieslawa Duszynska, Fabio Silvio Taccone, Magdalena Hurkacz, Beata Kowalska-Krochmal, Anna Wiela-Hojeńska, Andrzej Kübler

**Affiliations:** 1 Department of Anaesthesiology and Intensive Care, Wroclaw Medical University, Borowska Street 213, 50-556 Wroclaw, Poland; 2 Department of Intensive Care, Erasme Hospital, Route de Lennik 808, 1070 Brussels, Belgium; 3 Department of Clinical Pharmacology, Wroclaw Medical University, Borowska Street 213, 50-556 Wroclaw, Poland; 4 Department of Microbiology, Wroclaw Medical University, Chalubinskiego Street 4, 50-368 Wroclaw, Poland

## Abstract

**Introduction:**

Use of higher than standard doses of amikacin (AMK) has been proposed during sepsis, especially to treat less susceptible bacterial strains. However, few data are available on drug concentrations during prolonged therapy and on potential adverse events related to this strategy.

**Methods:**

Sixty-three critically ill patients who required AMK administration for the treatment of severe infection were included in this study. After a loading dose (LD, 18 to 30 mg/kg), the daily regimen was adapted using therapeutic drug monitoring (TDM) of both peak (C_peak_) and trough (C_min_) concentrations. Target concentrations had to give a ratio of at least 8 between C_peak _and the minimal inhibitory concentration (MIC) of the isolated pathogen. A C_min _>5 mg/L was considered as potentially nephrotoxic. We recorded clinical and microbiological responses, the development of acute kidney injury (AKI) during therapy and ICU mortality.

**Results:**

The median AMK LD was 1500 (750 to 2400) mg, which resulted in a C_peak_/MIC ≥8 in 40 (63%) patients. Increasing the dose in the 23 patients with a C_peak_/MIC <8 resulted in optimal C_peak_/MIC in 15 of these patients (79%). In 23 patients (37%), C_min _was >5mg/L after the LD, notably in the presence of altered renal function at the onset of therapy, needing prolongation of drug administration. Overall, only 11 patients (17%) required no dose or interval adjustment during AMK therapy. Clinical cure (32/37 (86%) vs. 16/23 (70%), *P *= 0.18)) and microbiological eradication (29/35 (83%) vs. 14/23 (61%), *P *= 0.07) were higher in patients with an initial optimal C_peak_/MIC than in the other patients. The proportion of patients with clinical cure significantly improved as the C_peak_/MIC increased (*P *= 0.006). Also, increased time to optimal C_peak _was associated with worse microbiological and clinical results. AKI was identified in 15 patients (24%) during AMK therapy; 12 of these patients already had altered renal function before drug administration. Survivors (*n *= 47) had similar initial C_peak_/MIC ratios but lower C_min _values compared to nonsurvivors.

**Conclusions:**

TDM resulted in adjustment of AMK therapy in most of our septic patients. Early achievement of an optimal C_peak_/MIC ratio may have an impact on clinical and microbiological responses, but not on outcome. In patients with impaired renal function prior to treatment, AMK therapy may be associated with a further decline in renal function.

## Introduction

Aminoglycosides are an important therapeutic option for the treatment of life-threatening infections in critically ill patients [1]. These drugs are usually used in combination with β- lactams in infections cause by Gram-negative bacteria to extend the spectrum of antimicrobial activity [1,2], and remain one of the few available therapies against multidrug-resistant pathogens, such as those producing extended-spectrum β-lactamases [3]. Recent guidelines on the treatment of sepsis have recommended using combination therapy, especially in patients with septic shock or if *Pseudomonas aeruginosa *is suspected [4,5]. In a meta-analysis, Kumar *et al*. also suggested that combination therapy including aminoglycosides increased survival rates among patients suffering from septic shock of various etiologies and with an expected mortality exceeding 25% [6].

The antibacterial activity of aminoglycosides is related to the peak concentration (C_peak_) obtained after drug injection [7]. In particular, a ratio between C_peak _and the minimal inhibitory concentration (MIC) of the pathogen of greater than eight has been associated with increased clinical cure in uncomplicated infections [8]. Nevertheless, sepsis may significantly alter the pharmacokinetic (PK) properties of these drugs, with increased distribution volume (Vd) and altered clearance, resulting in insufficient drug concentrations for the empiric treatment of *P. aeruginosa *[9,10]. In this respect, a higher than recommended loading dose (LD) of amikacin (AMK) was necessary to rapidly achieve therapeutic drug concentrations in patients with severe sepsis and septic shock [11]. However, the clinical impact of this strategy has not yet been studied among critically ill patients. Moreover, as these patients often suffer from acute kidney injury (AKI), which contributes to drug accumulation, the use of higher than recommended dose regimens may result in elevated trough concentrations (C_min_), which are associated with an increased risk of toxicity [12].

Galvez *et al*. showed that a daily dose of at least 30 mg/kg of AMK was necessary to achieve optimal drug concentrations [13]; no increase in renal toxicity was reported compared to lower dose regimens, including 15 and 25 mg/kg. However, overall mortality in this study [13] was only 9% and one may argue that these patients did not really represent a critically ill population with different organ dysfunctions and poor outcomes. Moreover, in such patients, the use of large amounts of fluid, of vasopressors and/or of renal replacement therapy may significantly impact on PK drug changes over time, which would be unpredictable if therapeutic drug monitoring (TDM) is not routinely performed [14].

The aim of this study was, therefore, to evaluate the impact of an AMK regimen based on dose adjustment using daily TDM in an ICU population with severe sepsis and septic shock.

## Materials and methods

### Patients

The study was conducted in the 20-bed ICU of the University Hospital in Wrocław, Poland, between May 2009 and May 2012. The University Bioethics Committee approved the study protocol; written consent was waived because TDM of aminoglycosides is routine practice in our ICU. All consecutive adult (>18 years) patients with a diagnosis of severe sepsis or septic shock according to standard criteria [15] were included if: a) the initial antibiotic therapy, other than amikacin, was appropriate (that is, the pathogen was sensitive *in vitro *to the first-line therapy); b) the attending physician decided to initiate AMK therapy. Drug regimens for other antibiotics were adapted to renal function according to standard recommendations. Exclusion criteria were: neutropenia (<1000/mm^3^), burns, myasthenia gravis, pregnancy, body mass index (BMI) >40 kg/m^2^, chronic renal failure requiring dialysis (HD) or the use of continuous renal replacement therapy (CRRT) at the onset of therapy.

### Infection diagnosis

The site of infection was diagnosed according to the CDC/NHSN criteria [16]. The protected non-bronchoscopic method (mini-bronchoalveolar lavage (BAL)) was used for microbiological diagnosis of ventilator-associated pneumonia (VAP), with a cutoff of at least 10^4 ^cultures/mL [17]. All biological samples were analyzed qualitatively and quantitatively according to established microbiological diagnostic criteria [16]. For each isolate, drug susceptibility tests (Etest, bioMérieux, Marcy l'Etoile, France) were performed to identify the MIC.

### Amikacin regimen and drug measurement

Amikacin therapy was combined with a first-line antibiotic, such as a β-lactam, sulphonamide or quinolone, when the Etest confirmed the presence of susceptible pathogen with an MIC ≤16 mg/L for the drug. Amikacin could be administered either: a) at the same time as the first-line therapy in case of previously colonization and an already known MIC; or b) on the second day of first-line therapy, when the MIC was available through the Etest from positive microbiological samples. The initial AMK (Biodacin; Bioton, Warsaw, Poland) dose was calculated using the ideal body weight (IBW), according to the Devine formula [17], for patients with a BMI between 18 and 28. In patients with a BMI <18, the total body weight (TBW) was used to calculate the AMK regimen. If the BMI was greater than 28 kg/m^2^, the dose was calculated using the adjusted body weight (ABW), which was calculated as: ABW = IBW + 0.4 (TBW-IBW) [18].

Immediately after the end of the LD (30 minutes, diluted in 100 mL of 0.9% NaCl), a 4-ml blood sample was taken to assess drug concentrations (C_max_). For each of the subsequent doses needing TDM, blood was sampled 30 minutes after the end of the dose for C_peak _and just before the next administration for C_min_. Blood samples were immediately sent to the laboratory for drug concentration measurement, using a validated qualitative and quantitative enzyme multiplied immunoassay technique (EMIT) on the Viva-E™ analyzer (Siemens, Dublin, Ireland). These measurements were controlled daily using internal and external control systems. The limit of quantification (LOQ) was 0.8 mg/L. Drug concentrations were measured daily only if dose correction or prolongation was necessary or if there were changes in serum creatinine (sCr), otherwise TDM was performed every 2 days. Because drug elimination is almost entirely dependent on renal function, we *a priori *defined two subgroups of patients, according to the baseline creatinine clearance (CrCl, that is <50 mL/min or ≥50 mL/min) estimated by the Cockroft-Gault formula [19], for separate analyses.

### Amikacin dose adjustment and PK analysis

The LD was established from the Etest estimation of MIC; if the MIC was ≤4 mg/L, the LD was 18 to 24 mg/kg (D1), but for MICs of 4 to 16 mg/L the LD was 25 to 30 mg/kg (D2). After the LD, AMK was administered once a day for a maximum of 5 to 7 days. The target C_peak_/MIC was between 8 and 12, with C_min _≤5 mg/L. Potential toxicity of AMK was considered at a C_min _>5 mg/L. If the C_peak_/MIC was <8, the daily dose (DD) was increased by 25 to 30% to a maximum of 30 mg/kg. If the C_peak_/MIC was >12, the DD was reduced by 25 to 30% to a minimum of 7.5 mg/kg. If C_min _was >5 mg/L, the time between two administrations was prolonged to 12 hours (to a maximum of 72 hours) to allow another drug concentration measurement; following dose was given only when C_min _was ≤5 mg/L. The probability of attainment (PTA) of optimal C_peak_/MIC was calculated using the initial C_peak _for a wide range of MICs (0.5 to 16 mg/L). PK parameters were calculated using a one-compartment model (Kinetica 5.0; Thermo Fisher Scientific, Poch SA, Gliwice, Poland). The following parameters were obtained: total volume of distribution (Vd), half-life time (t_1/2_), the area under the curve (AUC) and drug clearance (CL).

### Clinical and microbiological end points

The main end points of this study were clinical efficacy, microbiological response and the development of AKI. Clinical efficacy assessment was conducted on day 7 after the initiation of AMK therapy and was based on clinical signs (body temperature, BT), laboratory parameters (white blood cell count, procalcitonin and C-reactive protein) and the regression of tissue-related radiological signs or microbiological samples. Clinical cure was defined as complete resolution of clinical signs (BT <37.5°C) and laboratory (reduction of procalcitonin or C-reactive protein of more than 80% compared to baseline)/microbiological abnormalities (absence of pathogens on different samples), with no further need for antibiotic therapy or source control. Clinical failure was defined as a persistence or progression of clinical symptoms of infection, initial recovery followed by deterioration, change in wider spectrum antibiotic therapy (escalation), increased radiological infiltrates and/or worsening of laboratory data. Eradication was defined as a negative culture on day 7 of therapy. Microbiological failure was defined as the persistence of pathogen(s) in laboratory samples or as the development of a new infection. AKI was defined according to the AKIN criteria, that is, an increase in sCr of ≥0.3 mg/dL and/or a decreased urine output (<0.5 ml/kg/h for more than 6 hours) during AMK therapy until 72 hours after drug discontinuation [20]. Need for renal replacement therapy (RRT) was also considered as AKI.

### Statistical analysis

Statistical analyses were performed using the SPSS 13.0 for Windows NT software package (SPSS Inc., Chicago, IL, USA). Descriptive statistics were computed for all study variables. Discrete variables were expressed as counts (percentage) and continuous variables as means ± standard deviation (SD) or median (25^th ^to 75^th ^percentiles). Differences between groups were assessed using a chi-square, Fisher's exact test, Student's *t *test, or Mann-Whitney *U *test, as appropriate. A *P *<0.05 was considered to be statistically significant.

## Results

Sixty-three patients were included in this study. The patients' characteristics are shown in Table 1. Amikacin was combined with carbapenems (*n *= 21), cephalosporins (*n *= 14), piperacillin/tazobactam (*n *= 11), colistin (*n *= 8), trimethoprim/sulfamethoxazole (*n *= 3), ampicillin/sulbactam (*n *= 2), or other drugs (*n *= 4). The most common site of infection was the lung (*n *= 32) and 23 patients (44%) had positive blood cultures. The isolated Gram-negative bacteria were: *P. aeruginosa *(*n *= 19), *Escherichia coli *(*n *= 9), *Acinetobacter baumanii *(*n *= 7), *Klebsiella pneumoniae *(*n *= 6), *Enterobacter cloacae *(*n *= 4), *Proteus mirabilis *(*n *= 3) and others (*n *= 13). In 41 patients, AMK was initiated together with the first-line therapy; in the 22 other patients, AMK was started on the second day of therapy. The median MIC for amikacin was 4 (1 to 16) mg/L. Septic shock occurred in 20 patients (32%), with an overall ICU mortality of 25%.

**Table 1:** Demographics and clinical data of patients on drug initiation (*n *= 63) and with regard to initial C_peak_/MIC ratio and loading dose (D1 = 18 to 24 mg/kg; D2 = 25 to 30 mg/kg)

	All patients (*n *= 63)	C_peak_/MIC ≥8 (*n *= 40)	C_peak_/MIC <8 (*n *= 23)	D1 (*n *= 39)	D2 (*n *= 24)
Female/Male, n	22 / 41	14 / 30	8 / 11	13 / 26	9 / 16

Age (years)	59 ± 16	58 ± 15	60 ± 18	59 ± 15	59 ± 18

IBW (kg)	65 ± 10	65 ± 9	64 ± 10	64 ± 10	66 ± 9

ABW (kg)	67 ± 12	68 ± 13	66 ± 12	67 ± 13	67 ± 10

APACHE II score	21 (19-25)	21 (19-24)	23 (18-27)	21 (15-31)	21 (12-27)

SOFA	10 (8-11)	10 (8-11)	9 (8-11)	10 (7-11)	9 (8-11)

Severe sepsis/septic shock, n	43 / 20	26 / 14	17 / 6	28 / 11	15 / 9

Mechanical ventilation, n (%)	63 (100)	40 (100)	23 (100)	39 (100)	24 (100)

Immunosuppression, n (%)	13 (21)	8 (20)	5 (22)	8 (21)	5 (21)

Concomitant nephrotoxic agents, n (%)					
*Vancomycin*	4 (6)	3	1	3	1
*Colistin*	8 (13)	5	3	5	3
*Amphotericin B*	-	-	-	-	-
*NSAIDs*	24 (38)	15	9	14	10
*Contrast medium*	11 (17)	8	3	7	4
*Diuretics*	15 (24)	10	5	9	6
*Others*	1 (2)	1	-	1	-

Type of infection, n (%)					
*CR-BS*	10 (16)	6	4	5	5
*VAP*	34 (54)	24	10	23	11
*HAP*	7 (11)	6	1	6	1
*Peritonitis*	6 (9)	3	3	3	3
*Others*	6 (9)	1	5	2	4

Temperature, °C	37.7 ± 1.0	37.9 ± 0.8	37.6 ± 1.1	37.7 ± 1.0	38.0 ± 0.7

White blood cells, n × 10^3^/mm^3^	12.7 (8.5-16.7)	12.6 (8.4-19.3)	12.8 (8.8-15.9)	13.6 (8.8-17.1)	12.1 (8.1-20.1)

C-reactive protein, ng/mL	121 (69-166)	108 (60-159)	139 (102-196)*	113 (76-159)	132 (98-194)^#^

Procalcitonin, ng/mL	1.2 (0.5-5.9)	0.7 (0.3-4.6)	1.8 (1.1-6.8)*	0.7 (0.3-5.5)	2.0 (1.2-7.9)^#^

Hematocrit, %	27.9 (25.7-30)	28.8 (25.8-31.6)	27.0 (25.5-29.2)	28.0 (22.8-39)	26.7 (22.3-38.1)

Serum proteins, g/dL	4.9 ± 0.7	4.5 ± 0.6	5.0 ± 1.0	5.0 ± 0.8	4.9 ± 0.8

Albumin, g/dL	2.2 ± 0.5	2.1 ± 0.4	2.3 ± 0.5	2.3 ± 0.6	2.1 ± 0.3

Serum creatinine, mg/dL	1.1 ± 0.8	1.1 ± 0.8	1.1 ± 0.7	1.1 ± 0.9	1.0 ± 0.7

Creatinine clearance, mL/min	67 (50-118)	81 (52-108)	65 (45-130)	80 (40-108)	64 (50-143)

ICU stay, days	50 (18-65)	37 (14-77)	46 (11-58)	48 (24-68)	47 (17-66)

ICU mortality, n (%)	16 (25)	11 (27)	5 (22)	8 (21)	8 (33)

Data are presented as counts (percentage), mean (± standard deviation (SD)) or median (interquartile range (IQR). *P <0.05 vs. C_peak_/MIC ≥8; ^#^*P*<0.05 vs. D1. C_peak_, peak concentration; MIC, minimum inhibitory concentration; D1, loading dose 18 to 24 mg/kg; D2, loading dose 25 to 30 mg/kg; IBW, ideal body weight; ABW, adjusted body weight; APACHE, acute physiology and health evaluation; SOFA, sequential organ failure assessment; CR-BSI, catheter-related bloodstream infection; HAP, hospital-acquired pneumonia; VAP, ventilator-associated pneumonia.

**Figure 1:**
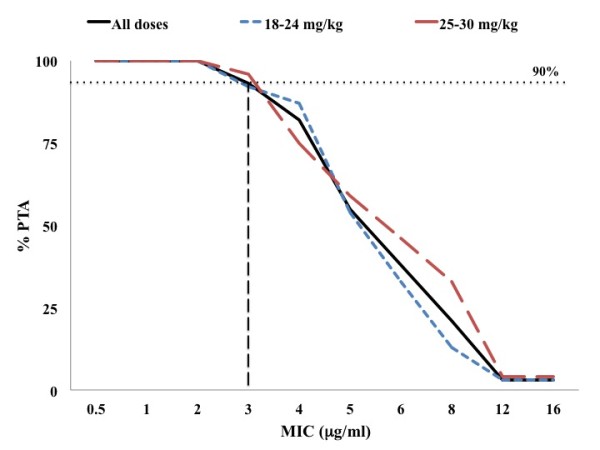
**Probability of attaining (PTA) the target C_peak_/MIC of ≥8 for various MICs when 18 to 24 mg/kg or 25 to 30 mg/kg dose regimens were administered**.

Median PK parameters for the LD were: volume of distribution 0.42 L/kg, total clearance 3.3 mL/min and half-life 5.4 hrs (Table 2). Median duration of therapy was 5 [3-6] days. The average AMK LD was 1500 (750 to 2400) mg; this resulted in a median C_peak _of 42.6 (20.3 to 142.0) mg/L and a C_peak_/MIC ≥8 in 40 (63%) patients. Patients with initial optimal C_peak_/MIC had similar characteristics than others, with the exception of lower C-reactive protein and procalcitonin on the first day of therapy (Table 1). A similar proportion of patients receiving D1 had an optimal C_peak_/MIC (26/39 (67%)) compared to those receiving D2 (14/24 (58%), *P *= 0.59). Among these 40 patients, 24 had C_peak_/MIC >12, requiring daily dose reduction (median 1200 (600 to 1800) mg). Only 13 patients (21%) had a C_peak _value greater than 64 mg/L, which corresponds to the target concentration to treat susceptible strains of *P. aeruginosa*. Notably, 5/39 (13%) patients receiving D1 and 8/24 (33%) receiving D2 achieved this target (*P *= 0.06). The PTA of optimal C_peak_/MIC was ≥90% for MIC ≤3 mg/L, regardless of the dose initially given (Figure 1). The daily dose was increased in the 23 patients (37%) with C_peak_/MIC <8 (median dose 2000 (1000 to 2400) mg), resulting in optimal C_peak_/MIC in 15 (79%) (Table 3).

**Table 2:** Pharmacokinetic parameters of amikacin loading dose according to the creatinine clearance (CrCl) at the onset of therapy

Parameter	All patients (*n *= 63)	CrCl ≥50 mL/min (*n *= 46)	CrCl <50 mL/min (*n *= 17)
**Vd (L)**	26.32 (8.4-51.7)	25.7 (8.5-49.3)	38.8 (8.4-46.7)

**Vd (L/kg)**	0.42 (0.12-1.03)	0.40 (0.12-1.03)	0.66 (0.2-0.8)*

**t_1/2 _(h)**	5.4 (0.8-19.9)	4.9 (0.8-11.0)	7.8 (3.2-19.1)*

**CL (mL/min)**	3.3 (0.6-25.3)	3.4 (1.5-25.8)	3.3 (0.6-9.9)

AUC_0-24 _(mg*h/L)	462 (58-1867)	361 (58-1045)	507 (153-1867)

Data are presented as median (ranges). *P = 0.05 vs. CrCl ≥50 mL/min. Vd, volume of distribution; t_1/2 _, half-life; CL, drug clearance; AUC_0-24_, area under the curve for the first day of therapy.

**Table 3:** Summary of dose adjustments during the study period

		Dose administration				
			
		24 hrs	36 hrs	48 hrs	72 hrs	
**Dose management **	Increased	14	2	4	3	23
	
	Unchanged	11	3	2	-	16
	
	Decreased	15	2	4	3	24

		40	7	10	6	63

In 23 patients (37%), C_min _was >5 mg/L after the LD, and the drug interval was increased (Table 3). Patients with CrCl ≥50 ml/min less frequently needed interval adjustment (8/43 (19%)) than those with CrCl <50 ml/min (15/20 (75%), *P *<0.001). There were no differences in the proportion of patients with C_min _>5mg/L between those receiving D1 and D2 (14/39 (36%) vs. 9/24 (38%), *P *= 0.93). On day 2 of therapy, 10 patients (15%) still had a C_min _>5mg/L, which returned below this threshold on day 3. Overall, only 11 patients (17%) required no dose or interval adjustment during AMK therapy.

**Figure 2:**
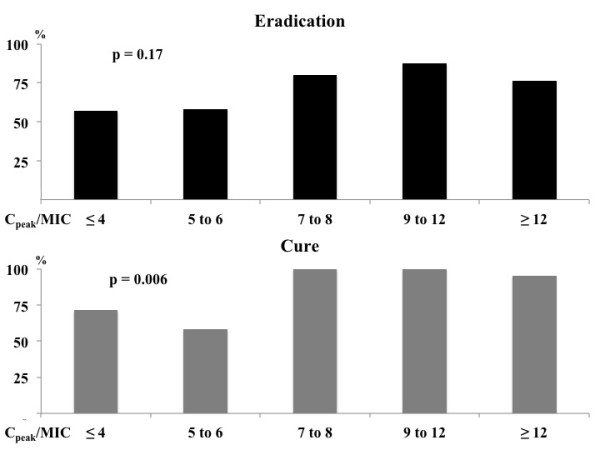
**Proportion of patients achieving microbiological eradication or clinical cure, according to different ratios of initial C_peak_/MIC**. Number of patients: C_peak_/MIC ≤4 (*n *= 7); C_peak_/MIC 5 to 6 (*n *=12); C_peak_/MIC 7 to 8 (*n *= 12); C_peak_/MIC 9 to 12 (*n *= 8); C_peak_/MIC >12 (*n *= 24).

**Figure 3:**
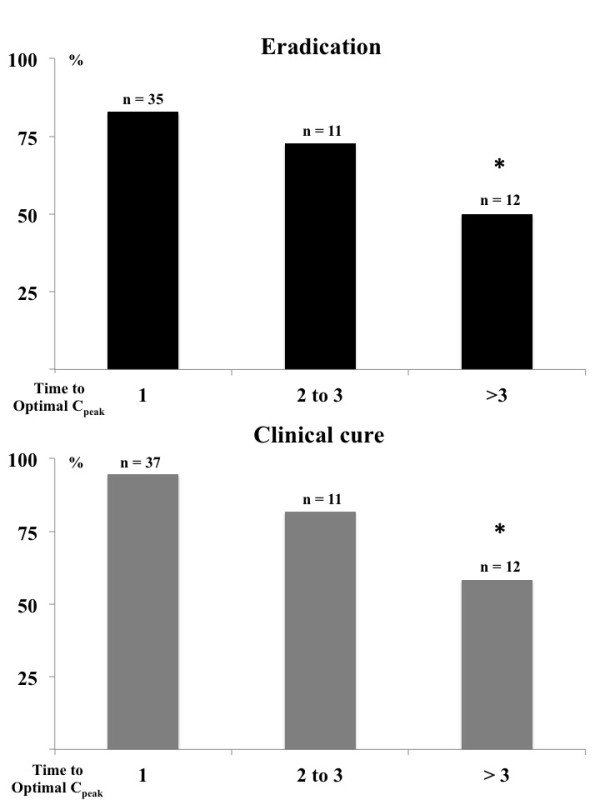
**Proportion of patients achieving microbiological eradication or clinical cure, according to the time (days) needed to achieve and C_peak_/MIC ≥8**. *P = 0.05 (eradication) and *P *= 0.01 (clinical cure).

Clinical cure was obtained in 51/60 (85%) patients and microbiological eradication in 43/58 (72%) patients; 3 patients could not be evaluated because they died before the end of therapy and 2 patients were weaned from mechanical ventilation and had no further microbiological samples taken to assess microbiological response. Specifically, clinical cure and microbiological eradication were achieved in 32/37 (86%) and 29/35 (83%) patients, respectively, with initial optimal C_peak_/MIC, compared to 16/23 (70%) and 14/23 (61%), respectively, patients with insufficient initial C_peak_/MIC (*P *= 0.18 and *P *= 0.07). The proportion of patients with clinical cure improved as the initial C_peak_/MIC increased (Figure 2). Moreover, patients with initial optimal C_peak _had significantly higher microbiological eradication and clinical cure rates than those achieving C_peak_/MIC ≥8 after more than 3 days of therapy (Figure 3).

There was no particular difference among patients receiving AMK as the first-line therapy compared to others for microbiological eradication (31/40 vs. 12/18, *P *= 0.51), clinical cure (35/41 vs. 15/19, *P *= 0.71) and mortality (10/41 vs. 6/22, *P *= 1.00). Similarly, patients with infections due to *P. aeruginosa *or *A. baumanii *(*n *= 26), had similar eradication (15/23 vs. 28/35, *P *= 0.23) and clinical cure rates (22/26 vs. 29/34, *P *=1.00) than others, but lower C_peak_/MIC (7.5 (5 to 11.5) vs. 13 (8 to 23.5), *P *= 0.005) and a trend toward fewer optimal C_peak_/MIC (13/26 vs. 27/37, *P *= 0.07). Nevertheless, this did not affect ICU mortality (5/26 vs. 11/37, *P *= 0.39, respectively). No particular differences were found when patients with respiratory infections (*n *= 41) were compared to others or when patients concomitantly treated with carbapenems (*n *= 21) were compared to those receiving other additional drugs (data not shown).

AKI developed in 15 patients (24%) during AMK therapy; among these, 12 patients had CrCl <50 ml/min at the onset of therapy and 6 of them eventually underwent RRT. The median time to develop AKI after the administration of the LD was 3 [2-6] days. Patients with AKI had similar C_peak _values to patients without AKI (46.8 (39.1 to 56.2) vs. 40.7 (33.8 to 53.9) mg/L, *P *= 0.44), but the same proportion of optimal initial C_peak_/MIC ratios (12/15 (80%) vs. 28/46 (61%), *P *= 0.27). C_min _values were higher (10.8 (5.1 to 15.5) vs. 1.8 (0.6 to 4.7) mg/L, *P *<0.001), initial CrCl was lower (44 (25 to 51) vs. 92 (65 to 132) mL/min, *P *<0.001) while total AMK dose was similar (3.9 (1.5 to 7.5) vs. 5.0 (2.5 to 15) g, *P *= 0.25) in patients with AKI than in those without. Survivors (*n *= 47) had similar initial C_peak_/MIC ratios (8 (6 to 18) vs. 13 (6 to 23), *P *= 0.26) than nonsurvivors, but lower C_min _values (1.8 (0.6 to 5.3) vs. 7.6 (3.6 to 13.5) mg/L, *P *= 0.002).

## Discussion

We have shown that higher than standard drug doses are initially necessary to obtain adequate C_peak_/MIC in critically ill septic patients. Moreover, TDM appears mandatory during aminoglycoside therapy, because only 17% of patients required no drug dose adjustment. Clinical cure and microbiological eradication occurred more frequently, although not statistically different, in patients with an optimal initial C_peak_/MIC than in other patients; however, there were no differences in ICU outcome between groups.

Because of widespread bacterial resistance to several groups of antimicrobials, AMK remains one of the more frequently prescribed aminoglycosides in critically ill patients, despite a very narrow therapeutic index and potential adverse events. Administration of a once daily dose of aminoglycosides in the treatment of severe infection has been widely accepted, because it achieves a higher C_peak _than multiple daily doses and is associated with a lower risk of AKI [7,12,21]. However, PK changes occurring during sepsis require the use of a higher AMK dose (25 to 30 mg/kg) to rapidly achieve optimal C_peak_/MIC, especially when treating less susceptible strains [11,13]. In burn patients, even higher dose regimens (38 mg/kg) have been proposed to optimize drug therapy [22]. In our study, 63% of patients achieved this PD target after the LD. These data are similar to those reported in recent studies on large ICU cohorts [11,13]. Nevertheless, important differences need to be underlined. First, we adapted the drug LD to the MIC of the pathogen, obtained before the onset of therapy. This approach was chosen to avoid the unnecessary risk of high AMK doses as we used this drug only for targeted therapy. When used as empirical therapy, drug regimens should be adapted rapidly to treat less susceptible strains, such as *P. aeruginosa *or *A. baumanii*, requiring a daily dose of at least 25 mg/kg and a C_peak _target above 64 mg/L. Second, even patients with an MIC of 8 to 16 mg/L received AMK, although according to EUCAST recommendations [23], these pathogens are resistant to the drug and optimal C_peak _can hardly be obtained in these situations. Although some authors have reported combining a very high AMK regimen (50 mg/kg) with high-flow CRRT to obtain adequate C_peak_/MIC and rapidly reduce drug concentrations below the threshold of potential toxicity [24], this approach needs to be further validated and the choice of another antimicrobial should be considered for strains with MICs 8 to 16 mg/L. Finally, we calculated drug regimens according to IBW/ABW and adjusted dose for underweight (BMI <18) or obese (BMI >28) patients [18,25]. Taccone *et al*. used a 25 mg/kg LD of AMK calculated using TBW; but the authors noted that there would have been a significant decrease in the proportion of patients with optimal C_peak _if this regimen had been used based on IBW [11]. Similarly, Galvez *et al*. reported that an AMK LD of 30 mg/kg resulted in a larger number of patients with optimal C_peak _than an LD of 25 mg/kg, if IBW was used to calculate drug dose regimens [13]. In our cohort, only 33% of patients receiving at least 25 mg/kg achieved optimal C_peak _values to treat less susceptible strains. These data suggest a relative underestimation of AMK doses in ICU patients when IBW is applied and the need for even higher doses (30 to 35 mg/kg) to improve drug efficacy. Importantly, the risk of excessive drug dosing in obese patients and the exact role of IBW- or ABW-guided dose prescription needs to be further studied.

Aminoglycosides have been used for decades. However, monotherapy is effective only in urinary tract infections, and meta-analyses have failed to show the superiority of aminoglycoside-β-lactam combination therapy compared to β-lactams alone [1]. However, aminoglycosides were usually given as multiple daily injections and no peak monitoring was performed to optimize the drug regimen. Currently, β-lactams remain the first-line therapy in severe sepsis and septic shock [4]; furthermore, combination with an aminoglycoside may improve patient outcomes, especially in the most severe cases [6]. To the best of our knowledge, this study is the first to report on the clinical and microbiological effects of using high doses of aminoglycosides to reach therapeutic C_peak _in critically ill septic patients. Recently, Delannoy *et al*. found that short-term combination therapy with an aminoglycoside for ICU-acquired bacteremias increased survival and reduced the duration of mechanical ventilation [26]. Our data suggest that early optimizing aminoglycoside peaks according to the MICs of the isolated pathogens may result in further increased therapeutic efficacy and in the clinical cure of severe infections.

If using higher than standard AMK doses is mandatory to optimize its antibacterial activity, the main concern is related to the potential development of renal damage. Aminoglycosides are eliminated by the kidneys and drug uptake by tubular cells is a saturable mechanism when drug concentrations exceed 15 μg/mL [27]. In our study, 24% of patients developed AKI, independent of the initial LD they received. Similarly, Gerlach *et al*. found AKI in 12% of critically ill surgical patients treated with AMK, especially if combined with other nephrotoxins [28]. In contrast, Galvez *et al*. found no increased risk of AKI between a drug regimen using 30 mg/kg compared to others using lower doses [13]. Clinical studies have suggested that AKI is more prevalent when there is pre-existing renal impairment or diabetes mellitus or in the case of prolonged therapy [27]. Our data also suggest that the risk of drug accumulation is dependent on baseline renal function, with higher C_min _values in patients with CrCl <50 mL/min. Indeed, 12/15 patients who developed AKI during AMK therapy already had reduced CrCl at the onset of therapy, making it difficult to separate the toxic effects of the drug from the kidney damage induced by sepsis. In patients without critical illness, the use of high aminoglycoside dose regimens did not result in AKI, provided that C_min _was monitored [29,30]. Nevertheless, ICU patients have several risk factors for AKI, including hypovolemia, shock, use of diuretics or contrast medium and the presence of chronic kidney disease prior to ICU admission [31], and aminoglycosides could further aggravate the development of renal dysfunction in this population. Among 360 consecutive patients treated with aminoglycoside therapy in an ICU, AKI occurred in 58% and was independently associated with a lower glomerular filtration rate, hypotension, a higher prevalence of diabetes and a more frequent association with other nephrotoxic drugs [32]. Simulation of the clinical effects of amikacin given once daily also showed that, for an MIC of 16 μg/mL and a desired cure rate of at least 90%, the probability of renal failure was expected to be 100% [33]. Thus, an increase in sCr should be expected among ICU patients with pre-existing renal dysfunction, high disease severity, and infections with pathogens with high MICs when high-dose aminoglycosides adapted to the optimal C_peak_/MIC are used.

Our study has some limitations. First, we did not evaluate the effectiveness of high vs. standard AMK doses. Although it is possible that this strategy would provide some benefits when compared to standard regimens, because of an increased postantibiotic effect and a potentially reduced risk of drug resistance development, we cannot draw any conclusions regarding the superiority of optimized peak concentrations in this setting. Second, we used the Cockroft-Gault formula to estimate renal function, although this approach has several limitations when applied to critically ill patients. Third, we could not demonstrate any survival benefits for those patients who rapidly achieved optimal C_peak_. This may be because of the higher proportion of optimal initial C_peak_/MIC ratios among patients who subsequently developed AKI, which may have blunted any potential beneficial effect of optimized therapy.

Moreover, even empirical aminoglycoside therapy with optimized C_peak _has never been demonstrated to improve outcome among critically ill patients. Further studies are needed to address this point in large ICU cohorts. Fourth, in one-third of the patients, AMK was initiated only when MIC was obtained by Etest. Thus, this could be considered as being in contrast to common recommendations, which suggest early administration of appropriate antibiotic therapy to improve survival rates in septic patients [6]. Nevertheless, the benefits of this approach have not been clearly demonstrated for combination therapy and this strategy would have resulted in patients with pathogens resistant to the drug being exposed to potentially undesirable adverse events of the drug without likely benefit. In addition, for patients without positive microbiological samples, no microbiological assessment would have been possible. Fifth, we could not provide any data on the appropriateness of the first-line therapy (that is β-lactams) concentrations, which could have been an additional determinant in the evaluation of microbiological eradication and clinical cure. Sixth, the ranges of delivered dose of AMK were quite large in each group. Thus, it is possible that more precise regimens (that is 20 mg/kg vs. 30 mg/kg) would have resulted in different results. Also, the quite limited number of patients included over a 3-year period was related to the strict inclusion and exclusion criteria; this may have introduced a selection bias in this study. Finally, we did not specifically calculate a sample size to estimate the number of patients to be included in this study, as we could not identify any previous data describing the effects of initial optimal C_peak _values on clinical and microbiological response in critically ill patients.

## Conclusions

TDM resulted in adjustment of AMK therapy in most of our septic patients. Early achievement of an optimal C_peak_/MIC ratio may have an impact on clinical and microbiological responses, but not on outcome. In patients with impaired renal function prior to treatment, AMK therapy may be associated with a further decline in renal function.

## Key messages

- Therapeutic drug monitoring was necessary to adjust amikacin dose in most septic patients.

- Early achievement of optimal C_peak_/MIC may have an impact on clinical and microbiological responses, but not on outcome.

- Pre-existing altered renal function is associated with development of AKI during amikacin therapy.

## Abbreviations

ABW: adjusted body weight; AKI: acute kidney injury; AKIN: acute kidney injury network; AMK: amikacin; APACHE: acute physiology and health evaluation; AUC: area under the curve; BAL: bronchoalveolar lavage; BMI: body mass index; BT: body temperature; CDC: Centers for Disease Control; CL: drug clearance; C_max_: maximum concentration; C_min_: minimum concentration; C_peak_: peak concentration; CrCl: creatinine clearance; CRRT: continuous renal replacement therapy; D1: loading dose 18 to 24 mg/kg; D2: loading dose 25 to 30 mg/kg; DD: daily dose; EMIT: enzyme multiplied immunoassay technique; EUCAST: European Committee on Antimicrobial Susceptibility Testing; IBW: ideal body weight; LD: loading dose; LOC: limit of quantification; MIC: minimum inhibitory concentration; NHSN: National Healthcare Safety Network; PD: pharmacodynamics; PK: pharmacokinetics; PTA: probability of target attainment; RRT: renal replacement therapy; sCr: serum creatinine; SD: standard deviation; SOFA: sequential organ failure assessment; t_1/2_: half-life; TBW: total body weight; TDM: therapeutic drug monitoring; VAP: ventilator-associated pneumonia; Vd: distribution volume.

## Competing interests

The authors declare that they have no competing interests.

## Authors' contributions

WD conceived the study protocol. AK, WD and AWH participated in the design and coordination of the study. WD and BKK supervised data collection. WD, FST, MH participated in data interpretation. WD and MH were responsible for drug adaption. WD and FST carried out the literature search. WD drafted the present manuscript. FST and AK revised the manuscript. All authors read and approved the final version of the manuscript.

## Funding

No funding sources.
